# Association of Sedentary Behavior and Physical Activity with Depression in Sport University Students

**DOI:** 10.3390/ijerph18189881

**Published:** 2021-09-19

**Authors:** Huixuan Zhou, Xiaotong Dai, Litian Lou, Chan Zhou, Wei Zhang

**Affiliations:** 1 Key Laboratory of the Ministry of Education of Activity and Physical Fitness, School of Sport Science, Beijing Sport University, Beijing 100084, China; daixiaotong@bsu.edu.cn (X.D.); 2669@bsu.edu.cn (L.L.); 2 School of Education, Beijing Sport University, Beijing 100084, China; 2019992718@bsu.edu.cn; 3 China National Institutes for Food and Drug Control, Beijing 102629, China

**Keywords:** depression, mental health, sedentary behavior, physical activity, sports university students

## Abstract

Background: Sports university students are usually expected to lead an active life and have a lower risk of depression. Therefore, there are few studies on depression and its risk factors among this population. This study aimed to investigate depression and its association with sedentary behavior and physical activity in sports university students. Methods: A cross-sectional survey was conducted among undergraduates majoring in physical education in a sports university in Beijing in March 2021. Students were asked about sociodemographic information, domain-specific sedentary behavior, physical activity, and depression (using a nine-item Patient Health Questionnaire). Chi-squared test and logistic regression were carried out to analyze the data. Results: Among a total of 584 participants, the detection rate of depression was 49.1%. The median of total sedentary time was 7.29 h per day. After adjusting for covariates, recreational screen time (OR = 1.540, *p* = 0.035), sedentary time spent completing schoolwork (OR = 0.658, *p* = 0.038), and participation in vigorous physical activity everyday (OR = 0.415, *p* = 0.001) and a few times per week (OR = 0.423, *p* < 0.001) were significantly associated with depression. Conclusions: Sports university students are not immune to depression and inactive lifestyles. Excessive recreational screen time may have an adverse effect on depression, which is somewhat independent of physical activity.

## 1. Introduction

University students are in a transitional period of growth, and the change of environment and the expectations of family and society for higher education imposes stress on university students, making some of them suffer from mental health problems. Depression is one of the common mental health problems among university students [1,2], which may have an adverse impact on their academic performance and their future development [3]. Various health behaviors have been indicated as risk factors of depression among this population. Due to the nature of the higher education system, sedentary behavior (SB) and lack of physical activity (PA) are some of them.

SB is defined as a person in a sitting or reclining posture requiring an energy expenditure lower than 1.5 metabolic equivalents [4]. A recent systematic review showed that university students spend 7.29 h per day in sedentary activity based on self-reported data [5]. Compared to the general young adult population, a considerable proportion of university students engage in higher levels of SB [5]. The SB levels of some university students even surpass those of desk-based workers [6]. Excessive SB has been found to be associated with an increased risk of depression according to a meta-analysis [7]. Among various SB domains, self-reported screen time has been found to be significantly associated with adverse mental health outcomes, while there is no significant association of mental health outcomes with sitting time spent at work and transportation [7,8].

PA has been shown to be associated with depression among university students [7,9,10,11,12,13,14]. Some evidence suggests that high levels of PA can play a protective role as a counter to the negative effects of SB on all causes of mortality [15], and on self-reported mental and social health outcomes in some regions [16]. However, some studies show that SB is a risk factor somewhat independent of PA for depression in university students [17,18], and for multiple adverse health outcomes in adults [7,19,20].

Sport university students are expected to lead an active lifestyle. High levels of PA and low levels of SB have been believed to be associated with a positive self-concept and reductions in mental health problems, such as depression and anxiety [21]. As a result, SB and PA in this population and their association with depression have received little attention. For studies focusing on this topic, the samples mainly originate from comprehensive university students [7,17,18]. Although some studies have conducted surveys on the psychological problems of university athlete students, analyses of related factors are dominated by sociodemographic factors [22,23,24,25]. Moreover, some systematic reviews showed that the prevalence of depression and other mental health symptoms during the COVID-19 pandemic was higher than before its outbreak in the pooled global population [26], and that university students in China suffered from a higher prevalence rate of depression during the pandemic than in normal circumstances [27]. Therefore, the depression of students of sports universities in China also warrants attention after the outbreak of COVID-19.

To this end, our study investigated depression, SB, and PA of students of a sport university and analyzed the association of depression with total and domain-specific SB and PA so as to provide a profile of depression and SB among this population and a reference for potential interventions in their lifestyle.

## 2. Methods

### 2.1. Participants

A cross-sectional survey was conducted on undergraduates majoring in physical education, a typical discipline in Beijing Sport University in March 2021, which included athlete students of physical education courses and non-athlete students of sport science courses. Undergraduates in grades 1 to 3 were asked to complete an online self-administered questionnaire. Senior students were excluded because they had been in internships and had not led a typical school life. 

### 2.2. Instruments

Measures for SB, PA, depression and demographic information were included in the online questionnaire.

Participants were asked to report their SB (h per day) across several domains: attending class, completing schoolwork, recreational screen time, socializing, performing hobbies, and any other sedentary activities over the last 7 days. 

PA was investigated by asking the frequency of participation in vigorous physical activity (VPA refers to activity requiring significant physical effort and making someone breathe much harder than normal). Participants indicated their participation level by checking one of the following categories: every day, a few times a week, a few times a month, a few times a year, and under no circumstances.

Depression was measured using a nine-item Patient Health Questionnaire (PHQ-9). The responses were divided into four levels according to the frequency of symptoms in the past two weeks: never = 0 points, several days = 1 point, more than half of the time = 2 points, and almost every day = 3 points. The total score of the scale was 0 to 27 points. Those scoring 5 and above had depressive symptoms [28]. This scale showed good validity in a previous study among Chinese university students [29,30]. The Cronbach’s alpha value of these nine items was 0.89, which was larger than 0.70 and showed good reliability in this survey [31].

Demographic information included gender, grade, profession, place of hometown, whether only child or not, parents’ marital status and monthly family income, body mass index (BMI), smoking and drinking. BMI was calculated based on self-reported weight and height (kg/m^2^). According to the Chinese Guideline for Overweight and Obesity Prevention, BMI ≥ 24 kg/m^2^ was defined as overweight. 

### 2.3. Procedure

The counselors were asked to provide the QR code for the online questionnaire in the WeChat group of each class. They provided and sent reminders a total of three times. Undergraduates completed the questionnaire anonymously and of their own free will. Informed consent was provided via the homepage of the online survey and was obtained when participants completed the survey. This study protocol was approved by the Ethics Committee of Sport Science Experiment, Beijing Sport University (2020046H).

### 2.4. Design and Data Analysis

The data were analyzed using the Statistical Package of the Social Sciences (SPSS Version 20; IBM Inc., Armonk, NY, USA). The median, inter-quartile range (IQR), mean, and standard deviation (SD) of total and domain-specific sedentary time were presented. Then, they were treated as dichotomous variables based on the median. The mean was used when comparing sedentary time with the other research reported in the Discussion section of this paper. The detection of depression, VPA, and demographic information was also treated categorically and was presented as number (%). The between-group differences were tested by the Chi-squared test (Bonferroni method). The association of depression with total and domain-specific SB and VPA was examined by a stepwise backward logistic regression, after adjusting for demographic information. Odds ratios (ORs) with 95% confidence intervals (CIs) were presented. The significance level was *p* < 0.05.

## 3. Results

### 3.1. Basic Characteristics of Participants by Depression

A total of 584 participants completed the questionnaire and were included in the analysis. The response rate was 33.4% among athlete classes and 74.3% among non-athlete classes. The basic characteristics of participants are shown in Table 1, stratified by the detection of depression. 

Most participants were male students (59.8%), 42.3% of the sample were freshmen, 61.8% were athlete students, 54.8% came from urban places, 57.9% were an only child in their family, 11% were overweight (BMI ≥ 24), 14.9% reported a smoking habit, and 61.6% reported a drinking habit (Table 1). 

Among the participants, the detection rate for depression was 49.1%. The depression rates among female students, junior students, non-athlete students, and students with divorced parents were higher than those among male students (*p* < 0.001), freshmen and sophomores (*p* = 0.036), athlete students (*p* = 0.005), and students with married parents (*p* = 0.042) (Table 1). 

### 3.2. Sedentary Behavior and Physical Activity

The time spent in SB is shown in Table 2. The median of total sedentary time was 7.29 h per day. For domain-specific SB, the median time was 2.14 h in attending class, 1.00 h in completing schoolwork, 1.43 h in recreational screen activities, 0.57 h in socializing, 0.29 h in performing hobbies, and 0.29 h in other sedentary activities. 

In the following analysis, the total and domain-specific SB were treated as dichotomous variables based on the median, and the frequency of participating in VPA was regrouped into three categories for the balance of distribution in each subgroup. 

The characteristics of the participants stratified by level of total sedentary time (≥7.29 h and <7.29 h) are presented in Table 3. Compared with females, non-athlete students, students from urban places, and students who were the only child in their family, there were higher proportions of male students, athlete students, students from rural places, and students with siblings reporting sedentary time less than 7.29 h per day (*p* < 0.001). Students from a high-income family (above 9000 CNY per month) were more likely to have longer sedentary time than students from families with lower incomes (less than 6000 CNY, *p* = 0.001). Students participating in VPA a few times a month or less were more likely to report longer sedentary time than students participating in VPA more frequently (*p* < 0.001).

Stratified by the detection of depression, the levels of SB and frequency of VPA of the participants are shown in Table 4. The participants who spent more than 2.14 h in class, more than 1.00 h recreational screen time, and had a total sedentary time of more than 7.29 h demonstrated higher depression rates than those who spent less class time (*p* = 0.009) and had less screen time (*p* = 0.008) and less total sedentary time (*p* = 0.036). Students participating in VPA a few times per month or fewer presented higher depression rates than those reporting more frequent VPA participation (*p* < 0.001).

### 3.3. Multivariate Regression Analysis of Depression, Sedentary Behavior, and Physical Activity

After adjusting for covariates, participants who spent longer on recreational screen time were more likely to be detected with depression (OR = 1.540, *p* = 0.035), while those spending more time completing schoolwork were less likely to be detected with depression (OR = 0.658, *p* = 0.038). Compared with students who participated in VPA a few times a month or less, those who participated in VPA every day (OR = 0.415, *p* = 0.001) and a few times per week (OR = 0.423, *p* < 0.001) were less likely to be detected with depression. The total sedentary time and time spent in attending class, socializing, performing hobbies, and other sedentary activities were not significantly associated with the detection of depression (Table 5).

## 4. Discussion

### 4.1. Detection of Depression in Students of a Sports University

In our study, the detection rate of depression among students of a sports university majoring in physical education was 49.1%. These results are higher in comparison with the overall detection rate of depression among Chinese university students (28.4%) [3], global university students (30.6%) [32] and medical students (27.2%) [33], presented in several systematic reviews and also higher than the results of some surveys on students from comprehensive universities in China (ranging from 29.7% in Guangdong [34] to 46.0% in Tibet [35]). However, the prevalence rate of depression in our study is lower than that in a survey on university students in China, which was measured during the pandemic (60.5%) [36]. 

Potential explanations for the prevalence of depression in this study are as follows. First, unexpected demands for both academic and athletic achievements may make students overwhelmed. Non-athlete students of sport science were required to master one sport skill in addition to a heavy academic burden. Athlete students have to withstand stress from physical training and competition [37]. Second, the COVID-19 pandemic may play a role in the depression symptoms of the participants. Guo et al. indicated that the COVID-19 pandemic has had a negative effect on the mental health of university students in China [27]. The cessation of competitive sport during and after the pandemic has increased the uncertainty in sport-related career development, which may put additional pressure on students of sports universities. However, the lockdown policy to counter the transmission of COVID-19 has seldom been implemented in China since May 2020, which may weaken the negative impacts of the pandemic on depression in university students in China. Therefore, the prevalence of depression in our study is relatively higher than those of studies conducted in normal circumstances but lower than that in the study conducted during the pandemic. Moreover, the selection of measures and investigation time may have had some effects on research findings. 

Anywise, our research findings suggested that the depression symptoms of students of sports universities should be taken seriously, and measures should be taken according to the results of risk factor analysis.

### 4.2. Sedentary Behavior of Sports University Students

It was found in our study that sports university students spent on average 7.70 h per day in total SB, 2.22 h in attending class, and 2.30 h recreational screen time, which were close to the results in global university students in a recent meta-analysis (total sedentary time: 7.29 h, time in class: 2.23 h, screen time on TV, computer and video games: 2.26 h) [5]. Nevertheless, sports university students spent less time completing schoolwork than global university students (1.25 h versus 2.85 h). 

Gender, PA, and obesity were potential correlates frequently examined by studies on university students. Our research found that, compared with female students, male students performed better in total sedentary time, which is consistent with the results of research among Ukrainian university students [38], freshmen in Tsinghua University in China [39], and students from a private university in Colombia [40]. However, there are a series of studies showing different results. For instance, Malmborg et al. [41] found that there was no significant difference in total sedentary time between male and female university students in Sweden, and Ruiz et al. [42] indicated that female students spent less time sedentary than male students in a Spanish university. The mixed results suggest that the correlation between gender and sedentary time varies from population to population.

There are mixed results regarding the interrelationship between SB and PA [16]. Our research showed that sports university students participating in VPA a few times a month or less spent more time in SB than those participating in VPA more than a few times a week. This result is consistent with the conclusion of a systematic review [43], and somewhat supports the theory that time spent in SB displacing time available for PA is the reason for the potential negative health effects of SB [21]. 

Our research found that being overweight was not associated with total sedentary time, which is contrary to most previous research [19,43]. The low proportion of overweight students in the sample from sports universities may have biased the results. Moreover, athlete students were found to spend less sedentary time than non-athlete students in our study, probably because they had a more intense training curriculum and are used to leading a more active life. Students who are an only child, from urban places, and have a wealthier family reported longer sedentary time, which indicated that the family environment may have some impact on SB, and the family members of students may need to be taken as a secondary target population in related interventions.

### 4.3. Association of Depression with Sedentary Behavior and Physical Activity

A meta-analysis of studies on the general population showed that SB is associated with an increased risk of depression, although the results of the individual studies were mixed [7]. In addition to the theory that SB may displace PA, which has been found to be beneficial to mental health [21], the underlying explanation also includes that prolonged sedentary time may prevent individuals from social interactions and hence increase their risk of depression [44]. Regarding domain-specific SB, excessive screen time has been found to be associated with an increased risk of depression among the general population [7]. However, recent calls have been made claiming that the use of screens may have some positive effects on health outcomes, depending on the purpose of screen use (e.g., educational vs. recreational, playing violent video games vs. active video games) [5,45,46]. However, evidence to support those calls is required, especially for the effects on mental health outcomes. Our study findings may shed some light on such views.

The results of the multivariate regression showed that recreational screen time was associated with an increased risk of depression among sports university students, while sedentary time spent completing schoolwork was associated with a decreased risk of depression; total sedentary time and other modalities of SB were not significantly associated with depression. Online education and paperless schoolwork have become increasingly popular among university students, especially after the breakout of the COVID-19 pandemic. Sports university students usually complete their schoolwork on computers or mobile phones. Our study indicated that sedentary time spent on educational purposes does not increase the risk of depression; time spent completing schoolwork may have a positive effect on reducing depression and only recreational screen time was associated with the increase in depression rate. Consistent with the view of some researchers [5,45], we suggest that rather than the total amount of sedentary time, the purpose of SB and screen use may be more important, and future investigations on SB and interventions targeting SB reduction among sports university students should pay closer attention to recreational screen time.

Moreover, our study showed that SB is a risk factor independent of VPA for depression among sports university students, which is consistent with most previous research taking depression as a health outcome [7,17,18]. Participating in PA at considerably higher than recommended levels may be able to decrease the mortality risk associated with SB [15]. However, our research suggested that frequent participation in VPA is associated with a decreased risk of depression, but is not sufficient to eliminate the adverse effects of excessive recreational screen time. Sports universities should pay attention to students’ mental health, and interventions targeting the reduction of recreational screen time and promotion of VPA should be warranted.

### 4.4. Limitations

This study has several limitations. First, the response rate in the questionnaire survey for athlete students was low, and the sample was from students in one sports university in Beijing. Therefore, the sample representative of sports university students needs to be considered. Second, the cross-sectional study design of this study could only analyze the association of depression with SB and PA, and could not make a causal inference. Third, the variables in our research were self-reported, which renders them vulnerable to subjectivity. The use of SB questionnaires is a developing field [18]. Generally, self-reported SB is lower than the results measured by an accelerator [5], but it is more applicable when investigating domain-specific SB. Fourth, according to the conclusions of previous studies [15,16], high levels of PA can play a role in combating the negative effects of SB on health outcomes, and, considering the length and acceptability of this self-administered questionnaire, our study only investigated the frequency of VPA to partly reflect high levels of PA in participants, instead of investigating PA using structured questionnaires. Therefore, the relationship of depression with mild to moderate PA was not explored in this study. Moreover, our study only focused on the detection of depression without investigating other psychological problems that may confront sports university students.

In the future, prospective cohort studies with a large-scale and more representative sample can be carried out on SB, PA, and psychological problems of sports university students to explore the causality between health behaviors and psychological problems and provide more evidence for the design of psychological interventions for this population.

## 5. Conclusions

By investigating depression, SB, and PA of sports university students, it was shown that depression is commonly found among this population, and their sedentary time is close to the level of global university students. Our results provide evidence of the association of depression with total and domain-specific SB by showing that only recreational screen time was associated with an increased risk of depression; educational sedentary time spent completing schoolwork was associated with a decreased risk of depression, and there was no relationship between depression and other modalities of SB. This study also supported that the effect of SB on depression is somewhat independent of the frequency of VPA. Sports university students are not immune to depression and inactive lifestyles. Their mental health and health behaviors require attention. Future research on the causal relationships between various reasons for SB, levels of PA, and depression are necessary for more targeted interventions among this population.

## Figures and Tables

**Table 1:** Characteristics of participants by depression, *n* (%).

Variable	Total	Depression	No Depression	*p*
**Gender**				<0.001
Male	349 (59.8)	150 (43.0)	199 (57.0)	
Female	235 (40.2)	137 (58.3)	98 (41.7)	
**Grade**				0.036
Freshman	247 (42.3)	112 (45.3)	135 (54.7)	
Sophomore	212 (36.3)	101 (47.6)	111 (52.4)	
Junior	125 (21.4)	74 (59.2) *	51 (40.8) *	
**Profession**				0.005
Athlete	361 (61.8)	161 (44.6)	200 (55.4)	
Non-athlete	223 (38.2)	126 (56.5)	97 (43.5)	
**Place of hometown**				0.902
Rural	264 (45.2)	129 (48.9)	135 (51.1)	
Urban	320 (54.8)	158 (49.4)	162 (50.6)	
**Only Child**				0.392
Yes	246 (42.1)	126 (51.2)	120 (48.8)	
No	338 (57.9)	161 (47.6)	177 (52.4)	
**Monthly family income**				0.666
Below 3000 CNY	102 (17.5)	54 (52.9)	48 (47.1)	
3000–5999 CNY	184 (31.5)	93 (50.5)	91 (49.5)	
6000–8999 CNY	124 (21.2)	56 (45.2)	68 (54.8)	
Above 9000 CNY	174 (29.8)	84 (48.3)	90 (51.7)	
**Parents’ Marital Status**				0.042
Married	533 (91.3)	255 (47.8)	278 (52.2)	
Divorce	51 (8.7)	32 (62.7)	19 (37.3)	
**Overweight**				0.083
Yes (BMI ≥ 24)	64 (11.0)	38 (59.4)	26 (40.6)	
No (BMI < 24)	520 (89.0)	249 (47.9)	271 (52.1)	
**Smoking**				0.055
Yes	87 (14.9)	51 (58.6)	36 (41.4)	
No	497 (85.1)	236 (47.5)	261 (52.5)	
**Drinking**				0.600
Yes	360 (61.6)	180 (50.0)	180 (50.0)	
No	224 (38.4)	107 (47.8)	117 (52.2)	
Total	584 (100)	287 (49.1)	297 (50.9)	

* Significant difference between subgroups.

**Table 2:** Sedentary time of participants (hours per day).

Sedentary Behavior	Median	IQR	Mean	SD
Attending class	2.14	1.36	2.22	0.89
Completing schoolwork	1.00	1.29	1.25	1.00
Recreational screen time	1.43	2.29	2.30	2.11
Socializing	0.57	1.29	0.92	1.06
Performing hobbies	0.29	0.71	0.52	0.74
Other	0.29	0.57	0.49	0.59
Total	7.29	5.93	7.70	4.09

IQR, inter-quartile range; SD, standard deviation.

**Table 3:** Characteristics of the participants by level of total sedentary time, *n* (%).

Variable	Total Sedentary Time per Day		*p*
	≥7.29 h	<7.29 h	
**Gender**			<0.001
Male	147 (42.1)	202 (57.9)	
Female	163 (69.4)	72 (30.6)	
**Grade**			0.442
Freshman	124 (50.2)	123 (49.8)	
Sophomore	115 (54.2)	97 (45.8)	
Junior	71 (56.8)	54 (43.2)	
**Profession**			<0.001
Athlete	139 (38.5)	222 (61.5)	
Non-athlete	171 (76.7)	52 (23.3)	
**Place of hometown**			<0.001
Rural	111 (42.0)	153 (58.0)	
Urban	199 (62.2)	121 (37.8)	
**Only Child**			<0.001
Yes	152 (61.8)	94 (38.2)	
No	158 (46.7)	180 (53.3)	
**Monthly family income**			0.001
Below 3000 CNY	42 (41.2)	60 (58.8)	
3000–5999 CNY	88 (47.8)	96 (52.2)	
6000–8999 CNY	69 (55.6)	55 (44.4)	
Above 9000 CNY	111 (63.8) *	63 (36.2) *	
**Parents’ Marital Status**			0.182
Married	238 (53.1)	250 (46.9)	
Divorce	27 (52.9)	24 (47.1)	
**Overweight**			0.083
Yes (BMI ≥ 24)	39 (60.9)	25 (39.1)	
No (BMI < 24)	271 (52.1)	249 (47.9)	
**Smoking**			0.330
Yes	42 (48.3)	45 (51.7)	
No	268 (53.9)	229 (46.1)	
**Drinking**			0.314
Yes	197 (54.7)	163 (45.3)	
No	113 (50.4)	111 (49.6)	
**Participating in VPA**			<0.001
Every day	56 (40.6)	82 (59.4)	
A few times a week	148 (48.8)	155 (51.2)	
A few times a month or less	106 (74.1) *	37 (25.9) *	

VPA, vigorous physical activity; * Significant difference between subgroups.

**Table 4:** Levels of sedentary behavior and frequency of vigorous physical exercise of participants by depression, *n* (%).

Variable	Total	Depression	No Depression	*p*
Attending class				0.009
≥2.14 h	310 (53.1)	168 (54.2)	142 (45.8)	
<2.14 h	274 (46.9)	119 (43.4)	155 (56.6)	
Doing schoolwork				0.914
≥1.00 h	310 (53.1)	153 (49.4)	157 (50.6)	
<1.00 h	274 (46.9)	134 (48.9)	140 (51.1)	
Recreational screen time				0.008
≥1.43 h	363 (62.2)	194 (53.4)	169 (46.6)	
<1.43 h	221 (37.8)	93 (42.1)	128 (57.9)	
Socializing				0.945
≥0.57 h	302 (51.7)	148 (49.0)	154 (51.0)	
<0.57 h	282 (48.3)	139 (49.3)	143 (50.7)	
Performing hobbies				0.821
≥0.29 h	333 (57.0)	165 (49.5)	168 (50.5)	
<0.29 h	251 (43.0)	122 (48.6)	129 (51.4)	
Other				0.895
≥0.29 h	258 (44.2)	126 (48.8)	132 (51.2)	
<0.29 h	326 (55.8)	161 (49.4)	165 (50.6)	
Total sedentary time				0.036
≥7.29 h	310 (53.1)	165 (53.2)	145 (46.8)	
<7.29 h	274 (46.9)	122 (44.5)	152 (55.5)	
Participating in VPA				<0.001
Every day	138 (23.6)	58 (42.0)	80 (58.0)	
A few times a week	303 (51.9)	132 (43.6)	171 (56.4)	
A few times a month or less	143 (24.5)	97 (67.8) *	46 (32.2) *	

VPA, vigorous physical activity; * Significant difference between subgroups.

**Table 5:** Association of depression with sedentary behavior and physical activity.

Variable	OR	Upper 95% CI	Lower 95% CI	*p*
Completing schoolwork (ref. < 1.00 h)				
≥1.00 h	0.658	0.442	0.978	0.038
Recreational screen time (<1.43 h)				
≥1.43 h	1.540	1.031	2.302	0.035
Participating in VPA (ref. a few times a month or less)				
Every day	0.415	0.242	0.712	0.001
A few times a week	0.423	0.270	0.662	<0.001

Adjusted for gender, grade, profession, place of hometown, only child or not, monthly family income, parents’ marital status, smoking and drinking; VPA, vigorous physical activity; OR, odd ratio; CI: confidence interval.

## Data Availability

The data presented in this study are available on request to qualified researchers from the corresponding author.
